# Brush swab as a noninvasive surrogate for tissue biopsies in epigenomic profiling of oral cancer

**DOI:** 10.1186/s40364-021-00349-x

**Published:** 2021-12-20

**Authors:** Chi T. Viet, Xinyu Zhang, Ke Xu, Gary Yu, Kesava Asam, Carissa M. Thomas, Nicholas F. Callahan, Coleen Doan, Paul C. Walker, Khanh Nguyen, Stephanie C. Kidd, Steve C. Lee, Anupama Grandhi, Clint T. Allen, Simon Young, James C. Melville, Jonathan W. Shum, Dan T. Viet, Alan S. Herford, Dylan F. Roden, Manuel L. Gonzalez, Jiang F. Zhong, Bradley E. Aouizerat

**Affiliations:** 1 Department of Oral and Maxillofacial Surgery, Loma Linda University School of Dentistry, Loma Linda, CA USA; 2 Department of Psychiatry, Yale School of Medicine, New Haven, CT USA; 3 VA Connecticut Healthcare System, West Haven, CT USA; 4 New York University Rory Meyers College of Nursing, New York, NY USA; 5 Department of Oral and Maxillofacial Surgery, New York University College of Dentistry, New York, NY USA; 6 Bluestone Center for Clinical Research, New York University College of Dentistry, New York, NY USA; 7 Department of Otolaryngology, University of Alabama at Birmingham, Birmingham, AL USA; 8 Department of Oral and Maxillofacial Surgery, University of Illinois Chicago, College of Dentistry, Chicago, IL USA; 9 Department of Otolaryngology, Loma Linda University School of Medicine, Loma Linda, CA USA; 10 Section on Translational Tumor Immunology, National Institute on Deafness and Other Communication Disorders (NIDCD), National Institutes of Health (NIH), Bethesda, MD USA; 11 Department of Oral, Head and Neck Oncology and Microvascular Reconstructive Surgery, School of Dentistry, University of Texas Health Science Center at Houston, Houston, TX USA; 12 Rocky Vista University, Ivins, UT USA; 13 Department of Otolaryngology, Rutgers New Jersey Medical School, Newark, NJ USA; 14 Department of Pathology, University of Alabama at Birmingham, Birmingham, AL USA; 15 Department of Basic Sciences, Loma Linda University, School of Medicine, Loma Linda, CA USA

**Keywords:** Methylation biomarker, Oral cancer, Head and neck cancer, Epigenetic biomarker, Brush swab, Brush biopsy, Biomarker, MethylCap-Seq, Methylation array

## Abstract

**Background:**

Oral squamous cell carcinoma (OSCC) has poor survival rates. There is a pressing need to develop more precise risk assessment methods to tailor clinical treatment. Epigenome-wide association studies in OSCC have not produced a viable biomarker. These studies have relied on methylation array platforms, which are limited in their ability to profile the methylome. In this study, we use MethylCap-Seq (MC-Seq), a comprehensive methylation quantification technique, and brush swab samples, to develop a noninvasive, readily translatable approach to profile the methylome in OSCC patients.

**Methods:**

Three OSCC patients underwent collection of cancer and contralateral normal tissue and brush swab biopsies, totaling 4 samples for each patient. Epigenome-wide DNA methylation quantification was performed using the SureSelectXT Methyl-Seq platform. DNA quality and methylation site resolution were compared between brush swab and tissue samples. Correlation and methylation value difference were determined for brush swabs vs. tissues for each respective patient and site (i.e.*,* cancer or normal). Correlations were calculated between cancer and normal tissues and brush swab samples for each patient to determine the robustness of DNA methylation marks using brush swabs in clinical biomarker studies.

**Results:**

There were no significant differences in DNA yield between tissue and brush swab samples. Mapping efficiency exceeded 90% across all samples, with no differences between tissue and brush swabs. The average number of CpG sites with at least 10x depth of coverage was 2,716,674 for brush swabs and 2,903,261 for tissues. Matched tissue and brush swabs had excellent correlation (*r* = 0.913 for cancer samples and *r* = 0.951 for normal samples). The methylation profile of the top 1000 CpGs was significantly different between cancer and normal samples (mean *p*-value = 0.00021) but not different between tissues and brush swabs (mean *p*-value = 0.11).

**Conclusions:**

Our results demonstrate that MC-Seq is an efficient platform for epigenome profiling in cancer biomarker studies, with broader methylome coverage than array-based platforms. Brush swab biopsy provides adequate DNA yield for MC-Seq, and taken together, our findings set the stage for development of a non-invasive methylome quantification technique for oral cancer with high translational potential.

**Supplementary Information:**

The online version contains supplementary material available at 10.1186/s40364-021-00349-x.

## Introduction

Each year 30,000 patients are diagnosed with oral cavity squamous cell carcinoma (OSCC), and unfortunately the incidence is on the rise [1–3].. Even for these early stage patients, the five-year survival rate is 60% [4]. Poor survival rates are in part due to inaccurate risk prediction. Early stage OSCC is primarily treated with surgical resection of the cancer, with or without adjuvant treatments such as an elective lymphadenectomy, radiation, or chemoradiation, for patients with high risk features. Currently, risk prediction to assign adjuvant treatment is entirely based on clinicopathologic information. Multiple retrospective and prospective studies have shown that these standard clinicopathologic factors have moderate accuracy with a concordance statistic (c-statistic) of 0.7 [4, 5]. The key to improving survival in OSCC lies in developing more accurate risk prediction methods, particularly in early stage patients. Although OSCC is a heavily epigenetically-regulated cancer [6], optimizing risk prediction using methylation features remains in its infancy. Methylation is one of the most frequent epigenetic changes in early oral carcinogenesis that is linked to cancer progression [6]. While several methylation studies in OSCC patients [6–17], including our own studies [7, 8], have highlighted differential methylation features between low and high risk patients, none of these studies have resulted in a clinically meaningful biomarker. Two main shortcomings of these previous studies are: 1) failure to use a clinically translatable array platform, and 2) failure to quantify methylation in real time, as cancer treatment is occurring.

With respect to the first challenge, the vast majority of methylation array studies in OSCC have used array-based platforms. While the Illumina Methylation 450 K or EPIC array are the most commonly used platforms for epigenome-wide association studies (EWAS), CpG site quantification is restricted at an upper limit of 870,000 sites, and results from these platforms have not been converted into a clinically-accessible risk prediction tool. Furthermore, the EPIC array content is frequently updated to enrich for cancer-associated genes, making comparison across cohorts challenging. Methylation capture sequencing (MC-seq) has a scalable workflow that can quantify methylation in a small subset of genes or the entire genome using next generation sequencing (NGS), with a higher likelihood of clinical translation due to broader CpG coverage in a more agnostic manner while maintaining its resolution in samples with modest DNA quantities [18].

With respect to the second challenge, clinical translation of a biomarker requires measurement at the onset of treatment in order to determine risk and the need for treatment escalation. Waiting until after cancer removal for the formalin-fixed, paraffin-embedded (FFPE) tissues would limit clinical translatability. The oral cavity has the advantage of being readily accessible for sampling, not only with tissue biopsies, but also with noninvasive techniques. Herein, we determine methylation features using noninvasive brush swabs. Brush swab biopsies of oral cancer and high grade lesions have been used in methylation analysis of a limited number of genes [19–21]. While the search for putative biomarkers is ongoing, this study focuses on the technological aspects of epigenome-wide profiling using noninvasive brush swab samples.

In this study, we hypothesize that brush swab biopsies serve as a robust noninvasive method to quantify cancer-specific methylation features. Using tissue and brush swab biopsies collected from OSCC patients at the time of surgery: 1) we determine the concordance between the methylation signature of cancer tissues and swabs vs. matched normal tissues and swabs using MC-Seq, and 2) we establish a workflow in which brush swabs and MC-seq are used at the time of diagnosis to establish a methylation signature that can be used to determine risk of mortality.

## Methods

### Patient selection and data collection

The patients were enrolled in a multi-institutional prospective clinical study in which biological samples and clinicopathologic information were collected. Collection of clinical data and samples was approved by the Institutional Review Board at each institution, which included Loma Linda University (LLU), University of Illinois Chicago (UIC), and University of Alabama at Birmingham (UAB). Patients were eligible if they were ≥ 18 years of age, had biopsy-proven squamous cell carcinoma of oral cavity sub-sites, including oral tongue, maxillary and mandibular gingiva, hard palate, floor of mouth, buccal mucosa, and lip mucosa, and no previous treatment of OSCC. Clinical and pathologic stages were recorded based on the American Joint Committee on Cancer (AJCC) Eighth Edition Staging Manual [22]. We collected the following information from the chart review: age, sex, race, smoking and alcohol use, staging, tumor location, pathologic characteristics, and treatment modalities received in addition to tumor ablation. Biological samples collected at the time of surgery include flash-frozen cancer and contralateral normal tissue, and brush swab biopsies of the cancer and contralateral normal site. Isohelix brush swabs (Boca Scientific) were brushed for a total of 20 times, with 10 times on each surface of the swab, at either the cancer or contralateral normal site. The brush swabs were preserved using 500ul BuccalFix™ stabilization solution (Boca Scientific). Samples were stored in − 80 °C. A total of 3 patients were randomly chosen from the ongoing prospective clinical study for the current study.

### Nucleic acid extraction and sample preparation

DNA was extracted from the flash-frozen tissue and brush swabs of the cancer and contralateral normal side of 3 patients, totaling 12 samples (4 samples per patient). Genomic DNA quality was determined by spectrophotometry and concentration was determined by fluorometry. DNA integrity and fragment size were determined using a microfluidic chip run on an Agilent Bioanalyzer.

### MC-seq target enrichment library prep

Indexed paired-end whole-genome sequencing libraries were prepared using the SureSelect XT Methyl-Seq kit (Agilent). Genomic DNA was sheared to a fragment length of 150–200 bp using the Covaris E220 system. Fragmented sample size distribution was determined using the Caliper LabChip GX system (PerkinElmer). Fragmented DNA ends were repaired with T4 DNA Polymerase and Polynucleotide Kinase and “A” base was added using Klenow fragment followed by AMPure XP bead-based purification (Beckman Coulter). The methylated adapters were ligated using T4 DNA ligase followed by bead purification with AMPure XP. Quality and quantity of adapter-ligated DNA were assessed with the Caliper LabChip GX system. Samples were enriched for targeted methylation sites by using the custom SureSelect Methyl-Seq Capture Library. Hybridization was performed at 65 °C for 16 h using a thermal cycler. Once the enrichment was completed, the samples were mixed with streptavidin-coated beads (Thermo Fisher Scientific) and washed with a series of buffers to remove non-specific DNA fragments. DNA fragments were eluted from beads with 0.1 M NaOH. Unmethylated C residues of enriched DNA underwent bisulfite conversion using the EZ DNA Methylation-Gold Kit (Zymo Research). The SureSelect enriched and bisulfite-converted libraries underwent PCR amplification using custom made primers (IDT). Dual-indexed libraries were quantified by quantitative polymerase chain reaction (qPCR) with the Library Quantification Kit (KAPA Biosystems) and inserts size distribution was assessed using the Caliper LabChip GX system.

### Flow cell preparation and sequencing

Samples were sequenced using 100 bp paired-end sequencing on an Illumina HiSeq NovaSeq according to Illumina protocol. A positive control (prepared bacteriophage Phi X library) was added into every lane at a concentration of 0.3% to assess sequencing quality in real time.

### Preprocessing and quality control

Signal intensities were converted to individual base calls during each run using the system’s Real Time Analysis software. Sample de-multiplexing was performed using Illumina’s CASAVA 1.8.2 software suite. The sample error rate was required to be less than 1% and the distribution of reads per sample in a lane to be within reasonable tolerance. Sequence data quality were examined using FastQC (ver. 0.11.8). Adapter sequences and fragments with poor quality were removed by Trim_galore (ver. 0.6.3_dev). Bismark pipelines (ver. v0.22.1_dev) were used to align the reads to the bisulfite human genome (hg19) with default parameters [23]. Sample alignment to the human genome was performed using bowtie 2 (ver. 2.3.5.1). Quality-trimmed paired-end reads were converted into a bisulfite forward (C- > T conversion) or reverse (G- > A conversion) strand read. Duplicated reads were removed from the Bismark mapping output and CpG extracted. All CpG sites were grouped by sequencing coverage (i.e., read depth); CpG sites with coverage ≥10x depth were retained for analysis to ensure high MC-Seq data quality. Genes were annotated using Homer annotatePeaks.pl. With this software, the promoter region is defined as 1 kilobase from the transcription start site (TSS).

### Comparison of methylation between tissue and brush swab biopsies

Benjamini-Hochberg FDR was applied to adjust *p* values per CpG site. Pearson correlations were calculated between tissue and brush biopsy samples of matched anatomic sites, and cancer and normal samples from the same patients. Pearson correlation and absolute difference were calculated among common CpG sites between the samples. Scatterplots were rendered showing the correlation of β values from all CpG sites measured by MC-seq. Separate scatterplots were rendered showing the concordance of these CpG sites between tissues and brush swabs for the cancer sites and the normal sites. Student t-tests were performed to compare β values between cancer and normal groups or tissue and brush swab groups. The most significant 1000 CpGs features in cancer vs. normal groups were selected. Based on these results the -log10(t-test *p*-value) was calculated for each of the 1000 CpG sites to compare the degree of divergence in the significance of the test statistics for these 1000 CpG between 1) cancer vs. normal and 2) tissue vs. brush swabs.

### Statistical analyses

Statistical analyses were performed in R environment (v. 4.1.0).

## Results

### Patient cohort characteristics and DNA quality

Clinicopathologic information for the 3 enrolled patients are detailed in Table 1. The 3 patients comprised both early and late stage OSCC (stage I and IV), as well as varying tobacco and alcohol consumption habits. Patients were 49 and 68 years old. Two patients were male and one was female. All patients were white, non-Hispanic. Cancer and contralateral normal tissue and brush swab biopsies collected at the time of surgery underwent DNA extraction, with the yield and quality shown in Table 2. With a total input volume of 30 μL for each sample, total input for tissue DNA ranged from 187 ng to 660 ng, and an average of 390 ng. Total input for swab DNA ranged from 51 ng to 1998 ng, with an average of 532 ng. The input range was consistent with our previous study demonstrating reproducible CpG site quantification using MC-Seq across this range [18]. In our previous study, DNA quantity as low as 150-300 ng and DNA quality comparable to the findings in Table 2 were successfully amplified using our workflow.

**Table 1:** Patient demographic characteristics

Patient	Age	Sex	Race	Tobacco use, pack years	Alcohol, drinks/wk	Site	TNM	Stage	Grade
**1**	68	F	White	Never	Never	Tongue	T1N0M0	I	Moderate
**2**	68	M	White	Former, 53	Former, 24	Tongue	T4aN0M0	IV	Moderate
**3**	49	M	White	Current, 72	Current, 14	Mandible	T4bN3bM0	IV	Moderate

**Table 2:** Characteristics of genomic DNA used as input for sequencing of tissue and brush swab biopsies

Sample	DNA concentration ng/μl	A260	A280	260/280	260/230	gDNA input, ng
1C swab	3.96	0.01	−0.004	−2.99	−0.18	118.8
1C tissue	22.00	4.45	2.210	2.01	2.18	660.0
1 N swab	1.70	−0.05	−0.048	0.95	0.33	51.0
1 N tissue	6.24	0.44	0.220	2.01	2.75	187.2
2C swab	4.24	0.01	−0.027	−0.27	−0.09	127.2
2C tissue	11.40	1.00	0.463	2.16	2.85	342.0
2 N swab	6.32	0.07	0.014	5.14	−1.45	189.6
2 N tissue	8.00	0.62	0.290	2.12	3.04	240.0
3C swab	66.60	1.82	0.971	1.87	2.86	1998.0
3C tissue	21.80	2.16	1.314	1.65	0.80	654.0
3 N swab	23.60	0.45	0.217	2.06	4.70	708.0
3 N tissue	8.48	0.39	0.191	2.05	5.66	254.4

### MC-Seq mapping efficiency assessment

**Fig. 1:**
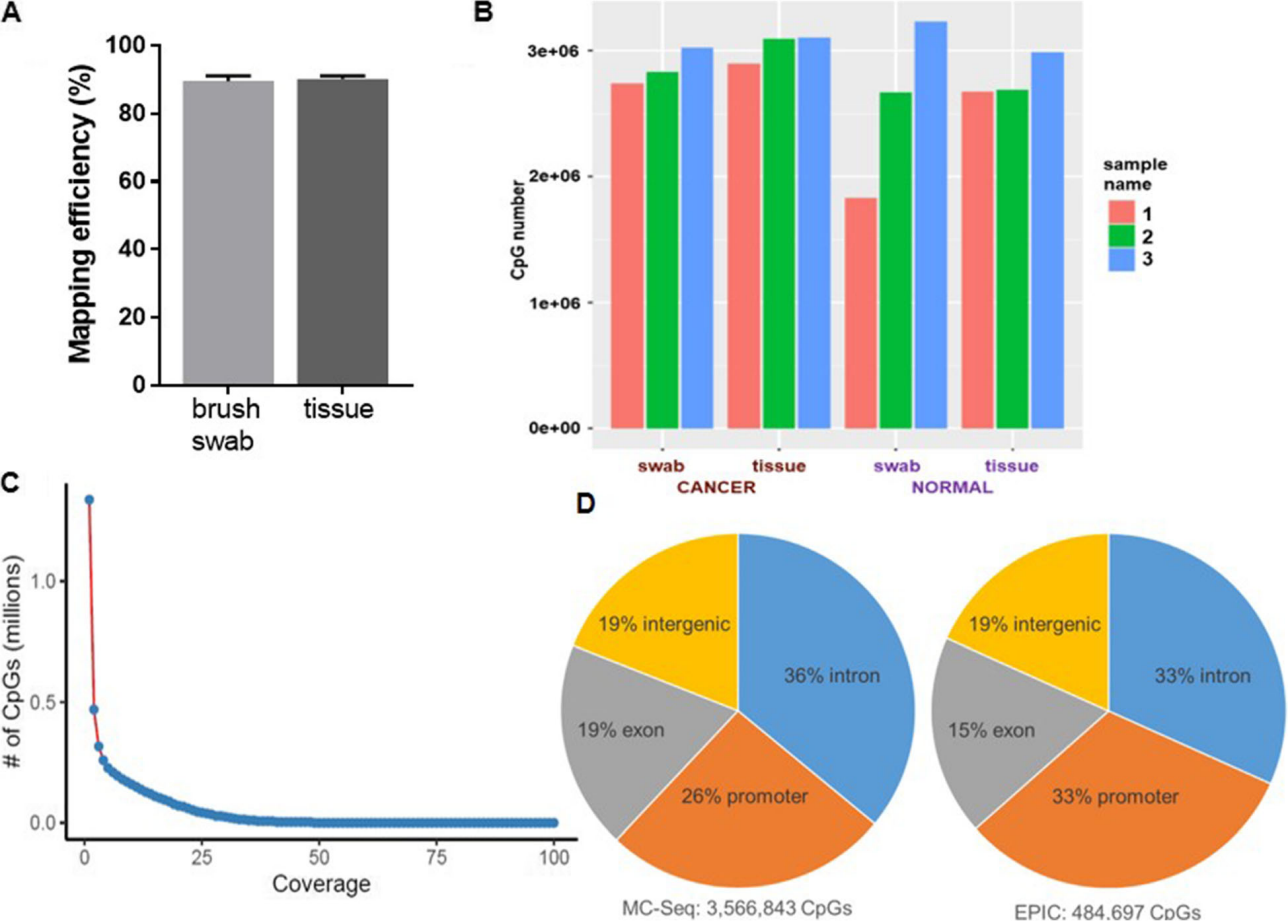
**(A)** We compared depth of coverage in all CpGs and determined an inflection point at 10x coverage. **(B)** Using 10x read depth as a cutoff, we determined the number of quantified CpG sites in each sample. Average number of quantified CpGs meeting our criteria was 2,716,674 for swab samples and 2,904,261 for tissue samples, with no significant difference between the two sample types. **(C)** The average mapping efficiency was 89.45% for brush swabs and 90% for tissues, with no significant difference between the two sampling methods. **(D)** The pie charts detail the relative genic locations of the CpGs profiled by MC-Seq (left) and CpGs covered by the EPIC array that were profiled (right). MC-Seq provided more robust coverage of functional gene regions than the EPIC array

Table 3 details the mapping efficiency for each biological sample. Using MC-Seq sequences mapped to the reference genome with an average mapping efficiency of 90% across all samples. There were no significant differences in mapping efficiency between tissues and brush swab samples (Fig. 1A). The average difference in mapping efficiency between the paired brush swabs and tissues was minimal, at − 0.567%, in favor of tissue samples, with a range of − 1.9 to 1.7%. The majority of methylated C’s appeared in a CpG context. We graphed the depth of read for each CpG across all queried CpGs and demonstrated an inflection point at 10x coverage (Fig. 1B). This finding was similar to our previous technical validation study, in which the majority of CpG sites exhibited at least 10x coverage [18]. We therefore applied this cutoff, focusing our analysis on CpG sites with at least 10x coverage. Average number of CpGs with at least 10x coverage was 2,716,674 for swab samples and 2,904,261 for tissue samples, with no significant difference between the two sample types, which is in excess of 3-fold greater CpGs interrogated than the most commonly used tool to measure the DNA methylome, the Illumina EPIC array. Figure 1C indicates the number of CpGs with at least 10x coverage for each of the 12 individual samples (see also Supplemental Figure 1).

**Table 3:** MC-Seq mapping efficiency among tissue and brush swab samples

Name	Mapping efficiency (%)	Difference between swab and tissue (%)	Sequence pairs analyzed in total	Number of paired-end alignments with a unique best hit	Duplicate (%)	Sequence pairs after removing duplicate	Total number of C	Total methylated C’s in CpG context	Total methylated C’s in CHG context	Total methylated C’s in CHH context	CpG with depth coverage > 10x
1C swab	90.7	0.1	42,303,352	38,369,629	52.22	18,333,433	749,965,581	37,720,278	2,408,697	5,995,678	2,738,093
1C tissue	90.6		34,732,207	31,480,976	28.42	22,534,549	927,088,792	44,803,115	2,908,059	7,258,418	2,892,873
1 N swab	89.3	−0.7	35,997,131	32,160,165	69.49	9,811,824	392,619,063	19,593,941	1,231,170	3,078,167	1,827,202
1 N tissue	90.4		38,013,580	34,357,686	50.99	16,840,023	701,455,939	36,205,354	2,320,490	5,789,438	2,671,771
2C swab	91.0	1.7	49,206,194	44,795,293	54.25	20,495,859	825,691,357	41,320,643	2,723,999	6,792,423	2,826,344
2C tissue	89.3		48,990,997	43,728,505	39.71	26,364,632	1,095,985,402	53,089,834	3,494,884	8,728,001	3,092,966
2 N swab	86.7	−1.9	40,037,338	34,712,643	48.22	17,975,067	745,570,397	35,629,449	2,300,915	5,877,073	2,662,761
2 N tissue	88.6		37,524,719	33,261,053	48.50	17,128,931	713,630,671	35,589,419	2,277,861	5,673,182	2,685,042
3C swab	90.7	−0.8	36,703,056	33,280,675	26.89	24,331,566	1,000,848,080	50,517,501	3,160,407	7,841,535	3,017,937
3C tissue	91.5		39,728,186	36,357,410	28.90	25,848,885	1,069,409,107	52,603,435	3,264,928	8,042,519	3,100,471
3 N swab	88.3	−1.8	52,334,030	46,226,126	34.76	30,156,313	1,221,133,676	62,639,138	3,840,377	9,388,785	3,227,707
3 N tissue	90.1		41,553,671	37,445,490	39.68	22,588,890	950,370,928	50,055,981	3,072,187	7,486,332	2,982,445
Average swab	89.45	−0.567	42,763,517	38,257,422	48.00	20,184,010	822,638,026	41,236,825	2,610,928	6,495,610	2,716,674
Average tissue	90.00		40,090,560	36,105,187	39.00	21,884,318	909,656,807	45,391,190	2,889,735	7,162,982	2,904,261

### Distribution of methylome regions

We determined the distribution of CpG sites profiled by MC-Seq among the CpG sites successfully measured at 10X depth of read or greater overlapping across all 12 samples (3,566,843 CpGs). Figure 1D demonstrates that 36% were in introns, 26% were in promoters, 19% were in exons, and 19% were in intergenic regions. Overall, MC-Seq provided more robust coverage of functional gene regions in the methylome than typically provided by the EPIC array, detecting ten-fold more CpG sites in promoter regions and exons than the EPIC array. We determined that 484,697 CpGs from the EPIC array, the majority of which were also found on the 450 K (396,409 CpG) were profiled by MC-Seq with at least 10x coverage. While the breakdown of these CpGs was 33% intron, 33% promoter, 15% exon, and 19% intergenic, the total number of CpGs in the functional gene regions was proportionally lower owing to the more limited coverage (Fig. 1D).

### Correlation between brush swab and tissue biopsies from matched anatomic sites

**Fig. 2:**
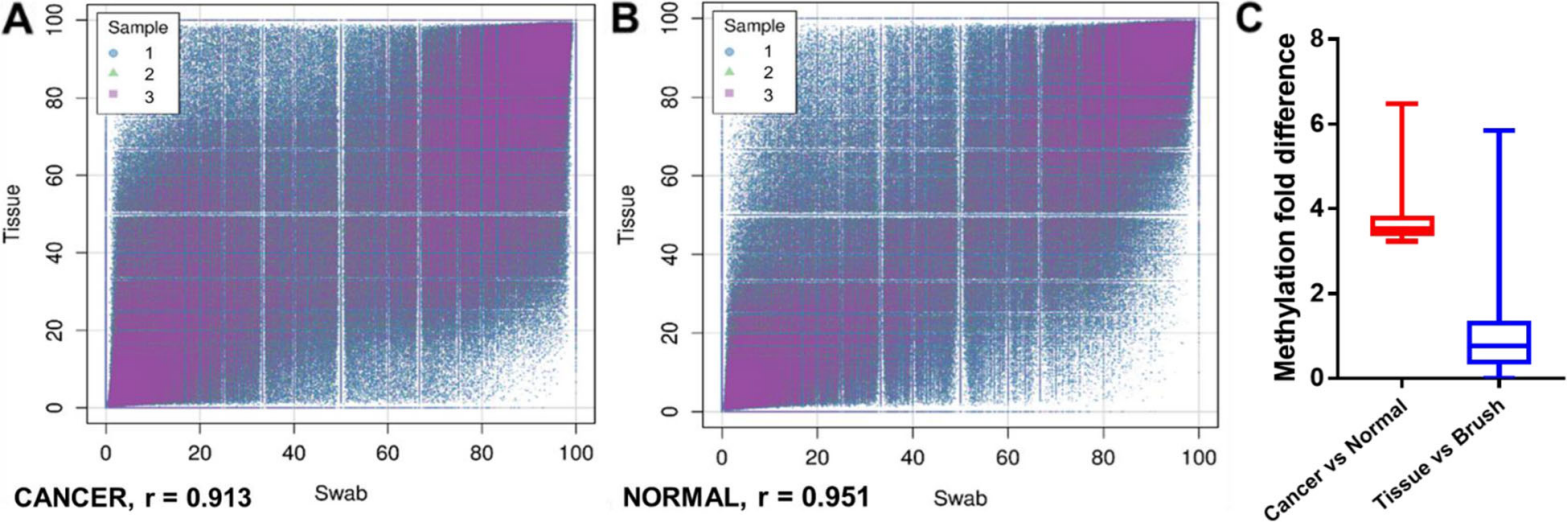
The scatterplots demonstrate the correlation between tissue and brush swab biopsies for **(A)** cancer and **(B)** normal sites of the 3 patients. The correlation values are noted. **(C)** By focusing on the top 1000 most variable methylation features between cancer and normal samples quantified with MC-Seq, we determined the methylation difference between different sample types, visualized using box plots (median, quartiles, maximum and minimum whiskers). The *p*-values for each test of difference in CpG methylation by t-test were expressed as -log_10_(*p*-value), which had a median of 3.67 (i.e., *p* = 0.00021) between cancer vs. normal. The same CpG sites were not differentially methylated [average -log_10_(*p*-value) = 0.96 (i.e., *p* = 0.11)] between tissue vs. brush swabs, suggesting that brush swabs are a viable surrogate of tissue biopsy

Overall, the correlation among CpG site methylation across all samples was high, all exceeding 90%. The average correlation between tissue and brush swabs (*n* = 12) among all CpG sites shared among the entire sample (cancer + control) (s = 3,566,843) was 93.2% (95% confidence interval: 93.23, 93.25%). The average correlation between tissue and brush swabs (*n* = 6) among all CpG sites shared among cancer samples was 91.3% (95% confidence interval: 91.32, 91.35%). The average correlation between tissue and brush swabs (*n* = 6) among all CpG sites shared among normal samples was 95.1% (95% confidence interval: 95.13, 95.14%). A scatterplot of the CpGs with 10x coverage was generated for the cancer samples and the normal samples separately, demonstrating high concordance between tissue and brush swabs (Fig. 2A and B).

### The top methylation features are differentially methylated between cancer and normal samples, but not between tissues and brush swabs

We focused on the top 1000 most variable methylation features between cancer and normal samples, which would be expected to differ considerably less between tissue and brush swab sampling methods. The *p*-values for each test of difference in CpG methylation by t-test were expressed as -log_10_(*p*-value), and averaged 3.67 (i.e., *p* = 0.00021) between cancer vs. normal. The same CpG sites were not differentially methylated, with an average -log_10_(*p*-value) = 0.96 (i.e.*, p* = 0.11) between tissue vs. brush swabs (Fig. 2C). The results suggest that brush swabs are a clinically viable surrogate for tissue biopsies.

## Discussion

### MC-seq is a scalable methylation assay that is currently not widely used in cancer research

EWAS studies in cancer patients have identified interindividual variability in the epigenome, and the recent availability of affordable EWAS technologies have led to a rapid increase in epigenetic biomarker studies aimed at identifying differential methylation features that could be predictive of clinical outcome. The most commonly used platforms are array-based, like the Illumina Human 450 K and Infinium MethylationEPIC arrays, which provide limited coverage of CpG sites across the epigenome. Whole genome bisulfite sequencing (WGBS) is the most comprehensive method for epigenome profiling, capturing 28 million CpGs. However, the cost, intensive workflow, and need for high quality and quantity of DNA input significantly limit its clinical translatability, particularly in cancer treatment. MC-Seq has emerged as a promising intermediary between arrays and WGBS, using NGS to capture significantly more CpGs than array-based platforms, while having the advantage of being more high-throughput and affordable than WGBS. We and others have compared CpG coverage and efficiency of different methylation quantification platforms [18, 24, 25]. A recent publication from our group has demonstrated that MC-Seq is a more reliable and efficient platform for epigenome profiling than array-based platforms like the EPIC array. When the EPIC array and MC-Seq were compared in peripheral blood mononuclear cell samples, MC-Seq captured significantly more CpGs in coding regions and CpG islands than the EPIC array. The EPIC array captured 846,464 CpG sites per sample, whereas MC-Seq captured 3,708,550 CpG sites per sample. Of the 472,540 CpG sites captured by both platforms, there was high correlation (*r* = 0.98–0.99) in methylation status [18]. Moreover, while the EPIC array is enriched for genes with known roles in carcinogenesis, MC-Seq quantifies methylation in a more agnostic manner and profiles 3–4 times more CpGs than the EPIC array, allowing for a higher chance of discovering novel epigenetic modifications in cancer. Furthermore, the coverage areas within each gene were more comprehensive than the EPIC array and other commonly used methylation analysis techniques, like PCR or pyrosequencing. Herein, we demonstrated that MC-Seq captured significantly more CpG sites within functional gene regions, owing to the higher overall profiling capability of this technique. The high throughput capabilities and depth of coverage make MC-Seq an appropriate, CLIA-approvable (Clinical Laboratory Improvement Amendments) platform to be used in a clinical setting.

### Oral SCC is an epigenetically-regulated cancer with promising methylation biomarker candidates

Methylation studies on OSCC patients [6–16] including our own studies [7, 8] have demonstrated that methylation is a common event and highlighted specific genes for mechanistic studies. For example, a EWAS using the Illumina Human 450 K array on 108 head and neck SCC patients of multiple sub-sites including oral cavity identified hypermethylation and inactivation of key tumor suppressor genes [9]. Clinical translation of these methylation biomarker studies has been limited due to: 1) combining OSCC with other head and neck cancer sub-sites (i.e.*,* oropharynyx, hypopharynx, larynx), which creates a heterogeneous cohort that fails to recognize OSCC as a distinct clinical disease, and 2) relying solely on array-based platforms, which query a limited number of CpGs. As a result, none of these studies have produced a methylation biomarker with high prognostic performance. We recently used methylation signatures combined with clinicopathologic data to develop a risk score to predict 5-year mortality of early-stage (I/II) OSCC; the risk score accurately predicted mortality with a c-statistic = 0.915 [5]. The risk score, which we named the REASON score, leveraged the top 12 differentially methylated genes between early-stage OSCC patients who survived vs. died at 5 years after diagnosis. Of note, 11 of the 12 genes had not previously been investigated in OSCC, with our study being the first to correlate differential methylation of these 11 genes with outcomes in OSCC [5].

In addition to being a distinct clinical subsite from other head and neck sites, the oral cavity is an easily accessible anatomic site for non-invasive biopsy techniques. Clinical translation of a biomarker requires that it can be measured during treatment. Waiting until after tumor removal for the formalin-fixed, paraffin-embedded (FFPE) tissues delays potentially necessary treatment. Researchers have used both saliva and brush swabs to noninvasively sample OSCC cells at the time of diagnosis. In our own studies, we have used saliva to identify methylation biomarkers of OSCC. We demonstrated that a multi-gene panel could be constructed using either a methylation array or Methylight, a polymerase chain reaction (PCR) technique [7, 8]. However, we and others have shown that concordance of methylation between saliva and cancer tissue is highly variable [26, 27].

### Brush swabs and MC-Seq represent a noninvasive method to quantify methylation biomarkers

Our approach of using brush swabs and MC-Seq to determine the methylation signature at the time of diagnosis has a high potential for clinical translatability. We demonstrated in this study that brush swab and tissue biopsies from matched sites had highly correlated methylation signatures. Furthermore, the DNA quality and quantity from brush swab samples were adequate to perform MC-Seq. Mapping efficiency was equivalent between tissues and brush swabs. Given the high correlation between the paired tissues and brush swabs, and the satisfactory DNA yield, brush swabs could serve as a clinically robust surrogate to tissue biopsies. One previous study has assessed the reliability of brush swab DNA for MC-Seq compared to the Human 450 K array [24], drawing similar conclusions to our study [18] that MC-Seq offered broader coverage of CpG sites and that sample-based correlation was high (*r* = 0.98) between the two platforms. However, they did not compare brush swab to underlying tissue collection. To our knowledge our study represents the first to directly compare the epigenome-wide signature of matched brush swabs and tissues, with the results having important implications in OSCC biomarker research. Our eventual goal is to apply our methylation risk score (REASON score) to a large cohort of patients, using brush swabs as a noninvasive method to determine methylation signatures for risk stratification.

## Conclusions

Our study establishes a workflow for a large-scale clinical study using brush swab samples and MC-Seq to noninvasively determine the methylation signature of OSCC patients at the time of diagnosis, which could be used to establish risk stratification schemes.

## Supplementary Information

**Additional file 1: Supplemental Figure 1.** The graphs demonstrate the M-bias coverage plots for each of the 12 samples

## Data Availability

All data generated for this study are included in this article.

## Abbreviations

EWAS: epigenome-wide association study
MC-Seq: MethylCap-Seq
OSCC: oral squamous cell carcinoma
