# Supplementary figures and images for: Brush swab as a noninvasive surrogate for tissue biopsies in epigenomic profiling of oral cancer
Source: Biomark Res. 2021 Dec 20;9:90. doi: 10.1186/s40364-021-00349-x (PMC8686381; doi:10.1186/s40364-021-00349-x)

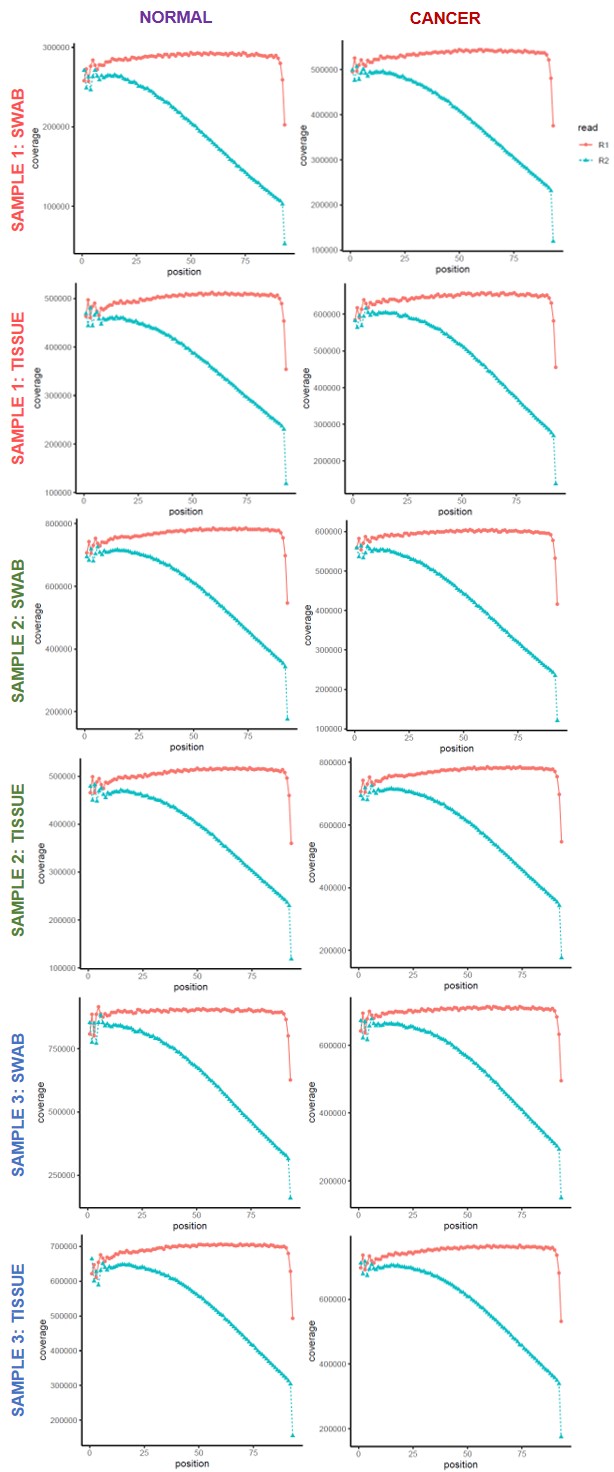

Supplement: Supplementary file 1 — Additional file 1: Supplemental Figure 1. The graphs demonstrate the M-bias coverage plots for each of the 12 samples [file 40364_2021_349_MOESM1_ESM.jpg]
